# Meat consumption and the risk of incident distal colon and rectal adenoma

**DOI:** 10.1038/bjc.2011.549

**Published:** 2011-12-13

**Authors:** L M Ferrucci, R Sinha, W-Y Huang, S I Berndt, H A Katki, R E Schoen, R B Hayes, A J Cross

**Affiliations:** 1 Division of Cancer Epidemiology and Genetics, National Cancer Institute, National Institutes of Health, Department of Health and Human Services, Bethesda, MD, USA; 2 Division of Chronic Disease Epidemiology, Yale School of Public Health, New Haven, CT, USA; 3 Department of Medicine, University of Pittsburgh, Pittsburgh, PA, USA; 4 Department of Environmental Medicine, New York University School of Medicine, New York, NY, USA

**Keywords:** colorectal adenoma, diet, meat, haeme iron, meat mutagens, nitrate, nitrite

## Abstract

**Background:**

Most studies of meat and colorectal adenoma have investigated prevalent events from a single screening, thus limiting our understanding of the role of meat and meat-related exposures in early colorectal carcinogenesis.

**Methods:**

Among participants in the screening arm of the Prostate, Lung, Colorectal, and Ovarian Cancer Screening Trial who underwent baseline and follow-up sigmoidoscopy (*n*=17 072), we identified 1008 individuals with incident distal colorectal adenoma. We calculated odds ratios (ORs) and 95% confidence intervals (95% CIs) for associations between meat and meat-related components and incident distal colorectal adenoma using multivariate logistic regression.

**Results:**

We observed suggestive positive associations for red meat, processed meat, haeme iron, and nitrate/nitrite with distal colorectal adenoma. Grilled meat (OR=1.56, 95% CI=1.04–2.36), well or very well-done meat (OR=1.59, 95% CI=1.05–2.43), 2-amino-1-methyl-6-phenyl-imidazo[4,5-*b*]pyridine (PhIP) (OR=1.75, 95% CI=1.17–2.64), benzo[*a*]pyrene (OR=1.53, 95% CI=1.06–2.20), and total mutagenic activity (OR=1.57, 95% CI=1.03–2.40) were positively associated with rectal adenoma. Total iron (diet and supplements) (OR=0.69, 95% CI=0.56–0.86) and iron from supplements (OR=0.65, 95% CI=0.44–0.97) were inversely associated with any distal colorectal adenoma.

**Conclusion:**

Our findings indicate that several meat-related components may be most relevant to early neoplasia in the rectum. In contrast, total iron and iron from supplements were inversely associated with any distal colorectal adenoma.

Consumption of red meat and processed meat has been positively associated with colorectal cancer in numerous epidemiological studies (WCRF/AICR, 2007); however, our understanding of whether these foods have a role during tumour initiation, progression, or both is limited. Few prospective epidemiological studies have been able to assess diet in relation to incident colorectal adenoma, a known precursor to colorectal cancer. This limited research on diet and incident colorectal adenoma has been primarily due to a lack of detailed data on screening procedures for colorectal cancer in prospective cohort studies, as the screenings are typically not conducted as part of these studies. In addition, randomised trials of colorectal adenoma prevention have evaluated recurrence of adenoma rather than incident adenoma. In colorectal adenoma prevention trials, processed meat (Robertson *et al*, 2005; Martinez *et al*, 2007), pan-fried meat (Martinez *et al*, 2007), and well/very well-done meat (Martinez *et al*, 2007) have been positively associated with recurrence of advanced or multiple adenoma, whereas dietary iron (Tseng *et al*, 1997) has been inversely associated with recurrence. However, the impact of diet on the carcinogenic process in individuals with a history of colorectal adenoma may differ from populations without a history of polyps and/or colon disease.

Thus far, most studies of diet, including meat and meat-related exposures, and colorectal adenoma have been for prevalent lesions discovered at a one-time screen (Tseng *et al*, 1996; Sinha *et al*, 1999, 2001, 2005c; Chan *et al*, 2005; Gunter *et al*, 2005; Shin *et al*, 2007; Ferrucci *et al*, 2009; Burnett-Hartman *et al*, 2011; Fu *et al*, 2011; Wang *et al*, 2011). Existing research on meat and incident colorectal adenoma is mixed and comes from five studies within just four different cohorts (Giovannucci *et al*, 1992; Tranah *et al*, 2004; Kesse *et al*, 2006; Wu *et al*, 2006; Rohrmann *et al*, 2009).

Several meat-related compounds may each contribute to colorectal neoplasia. Heterocyclic amines (HCAs) and polycyclic aromatic hydrocarbons (PAHs), meat mutagens formed during high-temperature cooking (Sinha *et al*, 1995, 1998a, 1998b), are gastrointestinal carcinogens in animal models (Ito *et al*, 1991; Ohgaki *et al*, 1991; Culp *et al*, 1998; Ochiai *et al*, 2002). Meat is also a source of iron, and both non-haeme and haeme iron can induce oxidative DNA damage (Glei *et al*, 2002; Tappel, 2007). Haeme iron, which is primarily found in red meat and is more bioavailable, has been associated with fecal water cytotoxicity (Sesink *et al*, 1999, 2000) and promotion of colorectal cancer in rodents (Pierre *et al*, 2004), as well as endogenous formation of *N*-nitroso compounds (NOCs) (Cross *et al*, 2003), powerful multi-site carcinogens (Bogovski and Bogovski, 1981; Lijinsky, 1992). Additional NOC exposure may occur from intake of nitrate and nitrite, precursors to NOCs that are added to processed meat for both preservation and colour.

With a lack of epidemiological research on the association between meat consumption and meat-related components and incident colorectal adenoma, we investigated this relationship in the screening arm of the Prostate, Lung, Colorectal, and Ovarian (PLCO) Cancer Screening Trial. Repeat flexible sigmoidoscopies conducted as part of the PLCO Cancer Screening Trial provided a unique context for studying adenoma as an incident rather than a prevalent event among a population free of distal colorectal adenoma at baseline, with adenoma status confirmed by follow-up sigmoidoscopy.

## Materials and methods

### PLCO Cancer Screening Trial

The PLCO Cancer Screening Trial is a multi-centre, randomised controlled trial designed to evaluate screening methods for the early detection of prostate, lung, colorectal, and ovarian cancer (Prorok *et al*, 2000). In brief, 154 952 participants (55–74 years of age) were recruited from 10 centres in the United States from 1993 to 2001 and randomly assigned to the screened or non-screened arm. Subjects underwent flexible sigmoidoscopy screening at baseline and then subsequently at either study year 3 or 5 (the protocol was modified in April 1995; changing from a 3- to 5-year interval). Lesions were not removed during trial sigmoidoscopies; subjects with abnormal findings were referred to their health-care providers for diagnostic follow-up. All participants completed a self-administered baseline questionnaire on demographics, personal and family cancer history, medical history, and lifestyle habits. Participants in the screening arm also completed a 137-item food frequency questionnaire (FFQ) on usual intake of foods and beverages during the past year (http://prevention.cancer.gov/files/programs-resources/dqx.pdf). Overall, 89% of participants completed the FFQ before or on the same day as the baseline sigmoidoscopy. Institutional Review Boards at the National Cancer Institute and the 10 study centres approved the study and participants provided written informed consent.

### Analytic population

These analyses were restricted to the screening arm of the trial (*n*=77 445). All participants had to have a normal sigmoidoscopy at baseline (i.e., no adenoma, no abnormal/suspicious findings) and not have had colorectal cancer before the baseline screen (*n*=49 500). Individuals with an inadequate (<50 cm) baseline sigmoidoscopy (*n*=7096) were considered ineligible. Among individuals who then underwent a PLCO follow-up (3 or 5 years after baseline) sigmoidoscopy (*n*=29 697), we excluded those who had colorectal cancer before or at the follow-up screen (*n*=16) or had an inadequate follow-up sigmoidoscopy (*n*=2915). Individuals who had a positive finding at follow-up sigmoidoscopy, but at the diagnostic endoscopy no adenoma was found (*n*=3370); individuals who had a positive finding at follow-up sigmoidoscopy, but a diagnostic endoscopy was not performed (*n*=418); and persons who had a positive finding at follow-up sigmoidoscopy, but the study was unable to obtain any information regarding diagnostic follow-up (*n*=460) were also excluded.

For these dietary analyses, we further excluded individuals who lacked either the baseline questionnaire (*n*=21) or the FFQ (*n*=2296) and those who missed >7 food items or reported energy intake in the top or bottom 1% on the FFQ (*n*=435) to remove people with implausible reported energy intake from the self-reported dietary data. Furthermore, we excluded participants who had a self-reported history of Crohn's disease, ulcerative colitis, familial polyposis, Gardner's syndrome, or colorectal polyps (*n*=1835), or a history of any cancer other than non-melanoma skin cancer before follow-up sigmoidoscopy (*n*=956). After these exclusions, our analytic population consisted of 17 072 individuals.

### Dietary exposures

Nutrient intake was estimated using the Diet^*^Calc Analysis Program (version 1.4.3, 2005, National Cancer Institute, Rockville, MD, USA), which is based on nutrient content information from the United States Department of Agriculture (USDA) Survey Nutrient Database and the Nutrition Data Systems for Research from the University of Minnesota. Intake (g per day) was calculated from frequency and portion size information. Red meat included beef, pork, and lamb products, whereas white meat encompassed poultry and fish. Processed meat included ham, hot dogs, liver, cold cuts, sausage, and bacon.

Questions on meat-cooking methods (barbequing, grilling, pan frying, and broiling) and doneness level (rare, medium, well, or very well done) for steak, bacon, sausage, pork chops, and hamburgers were included in the FFQ. With the Computerized Heterocyclic Amines Resource for Research in Epidemiology of Disease (CHARRED) (http://www.charred.cancer.gov) software application, we generated intake estimates of three HCAs (ng per day): 2-amino-3,4,8-trimethylimidazo[4,5-*f*]quinoxaline (DiMeIQx), 2-amino-3,8-dimethylimidazo[4,5-*f*]quinoxaline (MeIQx), and 2-amino-1-methyl-6-phenyl-imidazo[4,5-*b*]pyridine (PhIP), as well as benzo[*a*]pyrene (B[*a*]P), a marker of PAH exposure (Sinha *et al*, 2005a). Total mutagenic activity in meat (revertant colonies per day) determined by the standard plate incorporation assay with *Salmonella typhimurium* strain TA98 (Ames *et al*, 1975) was also estimated.

Meat-cooking data were used to estimate haeme iron intake from meat using a haeme iron database based on measured values from meat samples (Sinha *et al*, 2005a). Total iron (non-haeme and haeme) from meat (limited to those meats in the haeme iron database) was estimated from the nutrient content information in the USDA Survey Nutrient Database. Nitrate and nitrite intake from processed meats was calculated using a database of laboratory measured values of these compounds in 10 types of processed meats representing 90% of processed meats assessed by a typical FFQ in the United States (Sinha *et al*, 2005a).

### Outcome ascertainment

Incident distal colorectal adenoma cases were individuals with a positive follow-up sigmoidoscopy who then had a diagnostic follow-up endoscopy (e.g., colonoscopy, flexible sigmoidoscopy) conducted outside the study during which a distal colorectal adenoma diagnosis was confirmed. The PLCO personnel abstracted follow-up endoscopy records using a standard form that captured polyp histology, size, and location. Adenoma cases were restricted to those in the distal colon or rectum, as individuals with no abnormal/suspicious findings at follow-up sigmoidoscopy did not undergo diagnostic endoscopy. Cases were further classified into multiple distal colorectal adenoma (two or more adenoma) and advanced adenoma (size of ⩾1 cm, high-grade dysplasia, or villous components, including tubulovillous); with some individuals falling into both categories.

### Statistical analysis

Quartile cut points for dietary variables were based on intake of the analytic cohort. Odds ratios (OR) and 95% confidence intervals (95% CIs) were computed using unconditional logistic regression, with the first quartile as the referent group. Meat intake was nutrient density adjusted; using residual energy adjustment did not alter our findings (Willett, 1998). Total iron intake from diet and supplements was calculated by residually energy adjusting dietary iron and adding it to supplemental iron (mg per day). Multivariate models were adjusted for the following characteristics: age at baseline, study centre, gender, ethnicity, education, family history of colorectal cancer, body mass index, use of non-steroidal anti-inflammatory drugs, physical activity, smoking status, and intakes of alcohol, dietary calcium, supplemental calcium, dietary fibre, and total energy. To test for heterogeneity between the anatomical subsites (distal colon *vs* rectum), we calculated the weighted average of the two *β*-coefficients from the logistic regression model, with weights being proportional to the inverse of the variances. We then calculated the following *χ*^2^ statistic with one degree of freedom: 
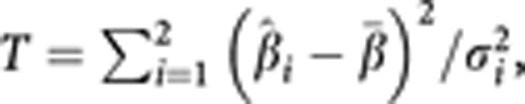
 where 
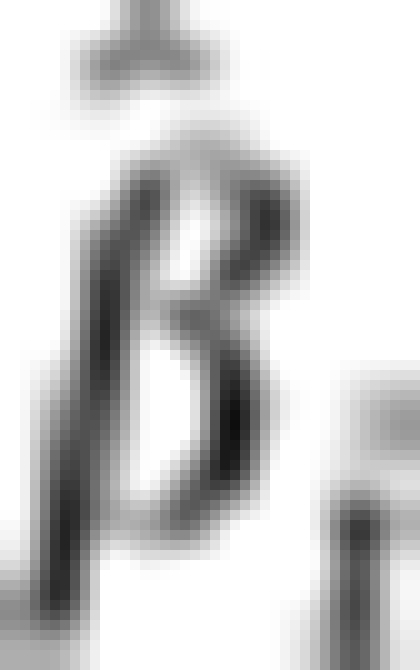
; and *σ*_*i*_^2^ are the coefficient and its variance for each subsite, and *β̂* is the weighted average of the *β*-coefficients. We also evaluated potential variation in risk estimates by gender, screening interval (3 or 5 years), and multiple and/or advanced adenoma. Trend tests were calculated using the median intake values of each quartile. *P*-values are two sided and analyses were conducted using SAS software (SAS Institute, Cary, NC, USA, version 9.2).

## Results

We identified 1008 individuals with incident distal colorectal adenoma 3–5 years after baseline, leaving 16 064 individuals with no abnormal/suspicious findings at both baseline and follow-up flexible sigmoidoscopies. Of the 1008 individuals with distal colorectal adenoma, 503 cases were non-advanced, 237 were advanced, and 268 were unknown for advanced status. A total of 131 individuals had multiple adenomas at follow-up and 772 had at least one adenoma in the distal colon, whereas 263 had at least one adenoma in the rectum (the specific location within the distal colorectum was unknown for 25 individuals). Individuals in the highest quartile of red meat were more likely to be non-Hispanic White and be current smokers compared with those in the lowest quartile of red meat consumption (Table 1). Participants who consumed the most red meat were also less likely to be female, less educated, and less physically active than those who consumed the least red meat.

We observed borderline statistically significant positive associations in the top quartiles of red (quartile 4 *vs* quartile 1 OR=1.22, 95% CI=0.98–1.52; *P*-trend=0.12) and processed (OR=1.23, 95% CI=0.99–1.54; *P*-trend=0.12) meat in relation to incident distal colorectal adenoma (Table 2). There was no heterogeneity between the risks observed for distal colon adenoma (OR=1.22, 95% CI=0.95–1.56) and rectal adenoma (OR=1.33, 95% CI=0.87–2.04) for red meat intake (*P*_heterogeneity_=0.73). In contrast, the risk estimate observed for processed meat and colon adenoma (OR=1.24, 95% CI=0.97–1.59) was higher than the risk estimate for rectal adenoma (OR=1.08, 95% CI=0.71–1.65), although this difference was not statistically significant (*P*_heterogeneity_=0.58). Risk estimates for advanced and/or multiple adenoma (323 cases) were not statistically significant for red (OR=1.32, 95% CI=0.91–1.94) or processed meat (OR=1.12, 95% CI=0.76–1.65). White meat intake was not associated with incident distal colorectal adenoma; there was also no clear association when separated into chicken (quartile 4 *vs* quartile 1 OR=0.95, 95% CI=0.78–1.15) and fish (OR=1.16, 95% CI=0.96–1.40).

There were some suggestive positive associations for grilled meat (OR=1.20, 95% CI=0.97–1.49), meat cooked well or very well-done (OR=1.20, 95% CI=0.95–1.49), and PhIP (OR=1.18, 95% CI=0.96–1.45) and colorectal adenoma for individuals in the top quartiles of intake compared with those in the lowest quartiles (Table 3). Associations for grilled meat (OR=1.56, 95% CI=1.04–2.36 *vs* OR=1.18, 95% CI=0.93–1.51; *P*_heterogeneity_=0.26), well or very well-done meat (OR=1.59, 95% CI=1.05–2.43 *vs* OR=1.14, 95% CI=0.89–1.46; *P*_heterogeneity_=0.18), and PhIP intake (OR=1.75, 95% CI=1.17–2.64 *vs* OR=1.07, 95% CI=0.85–1.36; *P*_heterogeneity_=0.04) seemed stronger for rectal adenoma than colon adenoma. Although there were no clear associations for mutagenic activity and B[*a*]P intake in relation to any distal colorectal adenoma, both exposures were positively associated with rectal adenoma (OR=1.57, 95% CI=1.03–2.40; OR=1.53, 95% CI=1.06–2.20, respectively), but not colon adenoma (OR=0.96, 95% CI=0.76–1.22; OR=0.92, 95% CI=0.74–1.15, respectively) with evidence of heterogeneity by subsite (*P*_heterogeneity_=0.05; *P*_heterogeneity_=0.02 for mutagenic activity and B[*a*]P, respectively). Analyses of advanced and/or multiple adenoma also revealed a positive association for well or very well-done meat (OR=1.45, 95% CI=1.01–2.09), but there was no variation by adenoma type for the other cooking variables or meat mutagens (data not shown).

Total iron intake was inversely associated with colorectal adenoma, as those who had the highest intake were 31% less likely to have an adenoma than were those with the lowest intake (OR=0.69, 95% CI=0.56–0.86, *P*-trend=0.01) (Table 4). Analysing dietary and supplementary iron separately revealed an inverse association for iron from supplements (OR=0.65, 95% CI=0.44–0.97, *P*-trend=0.02), and a similar, although not statistically significant, inverse association for dietary iron (OR=0.85, 95% CI=0.68–1.07, *P*-trend=0.06). In contrast, there was a borderline positive association between haeme iron from meat and colorectal adenoma in the top quartile of intake (OR=1.23, 95% CI=0.99–1.52), with evidence for a linear relationship (*P*-trend=0.03). We also observed a suggestive positive association for the highest intake of nitrite plus nitrate from processed meat compared with lowest (OR=1.22, 95% CI=0.94–1.53), although the linear trend was not statistically significant (*P*-trend=0.14). Associations for iron exposures and nitrate plus nitrite did not vary when we investigated advanced and/or multiple adenoma (data not shown) and there was little evidence for differences by anatomical subsite.

Interactions by gender were not statistically significant (*P*>0.05) (data not shown). In addition, we evaluated all associations stratified by time between baseline and follow-up flexible sigmoidoscopy screening (3 or 5 years) and risk estimates were not substantially different (data not shown).

## Discussion

In this comprehensive investigation of meat and meat-related compounds in relation to incident distal colorectal adenoma, we observed borderline positive associations for red meat, processed meat, grilled meat, well or very well-done meat, PhIP, haeme iron from meat, as well as nitrate and nitrite from processed meat. However, in analyses by anatomical subsite, there were statistically significant positive associations for rectal adenoma with grilled meat, well or very well-done meat, PhIP, B[*a*]P, and total mutagenic activity. We also found statistically significant inverse associations between total iron (diet and supplements) and iron from supplements and distal colorectal adenoma.

Although epidemiological evidence indicates red meat consumption is a risk factor for colorectal cancer (WCRF/AICR, 2007), studies of colorectal adenoma are not as clear with some positive associations for prevalent adenoma (risk estimates ranging from to 1.4 to 2.02 for highest *vs* lowest) (Sinha *et al*, 1999; Ferrucci *et al*, 2009; Fu *et al*, 2011), but also several null findings for both prevalent (Gunter *et al*, 2005; Sinha *et al*, 2005c; Shin *et al*, 2007; Burnett-Hartman *et al*, 2011; Wang *et al*, 2011) and recurrent adenoma (Mathew *et al*, 2004; Robertson *et al*, 2005; Martinez *et al*, 2007), with risk estimates ranging from 0.88 to 1.19. Studies of meat and incident colorectal adenoma are similarly inconclusive. There was a positive association in men between the ratio of red meat to white meat and incident adenoma of the distal colon and rectum (RR=1.83, 95% CI=1.12–3.00) (Giovannucci *et al*, 1992); however, with the accrual of additional cases, an analysis of adenoma of the distal colon only found positive associations for processed meat (RR=1.52, 95% CI=1.12–2.08), but no association with red meat (RR=1.18, 95% CI=0.87–1.62) (Wu *et al*, 2006). Among women, a Western dietary pattern high in meat was associated with incident distal colorectal adenoma in a French cohort (RR=1.39, 95% CI=1.00–1.83) (Kesse *et al*, 2006). Red and processed meat combined were also associated with colon, but not rectal, adenoma in a sample of both men and women (RR=1.63, 95% CI=1.01–2.30) (Rohrmann *et al*, 2009). Finally, in a subset of the incident distal colorectal adenoma cases (*n*=356) and controls (*n*=396) from the PLCO Cancer Screening Trial, red meat was associated with a 59% statistically significant increased risk of disease (Cross *et al*, 2011), but this was not replicated in the full sample analysed here. As other populations have not had access to colorectal screening results for all participants, instead relying on self-report of negative adenoma status as screenings were not a part of the study, these previous studies may suffer from false-negative reporting among controls. Similarly, adenoma-free status at baseline for both cases and controls in previous analyses was determined solely by self-report. These studies have been further limited by a low number of cases (<600) reducing the power to detect modest associations.

Although there were only suggestive associations for meat-cooking methods, doneness level, and meat mutagens in relation to any distal colorectal adenoma in our population, we observed a statistically significant elevated risk of rectal adenoma with high levels of grilled meat, well or very well-done meat, PhIP, B[*a*]P, and total mutagenic activity. Several studies have observed positive associations between intake of well-done meats or meats cooked by high-temperature methods and colorectal cancer (Gerhardsson de Verdier *et al*, 1991; Sinha and Rothman, 1999; Le Marchand *et al*, 2002; Butler *et al*, 2003; Murtaugh *et al*, 2004) and prevalent (Sinha *et al*, 1999, 2005c; Gunter *et al*, 2005; Fu *et al*, 2011) or recurrent (Martinez *et al*, 2007) colorectal adenoma. There is limited and inconclusive evidence on meat mutagens in relation to colorectal cancer, as some studies have found positive associations with colorectal cancer (risk estimates ranging from 1.16 to 4.09) (Kampman *et al*, 1999; Nowell *et al*, 2002; Butler *et al*, 2003; Cross *et al*, 2010), yet others have been null (Murtaugh *et al*, 2004) or inverse (Augustsson *et al*, 1999), with risk estimates ranging from 0.60 to 0.89. Results from prevalent and recurrent adenoma studies point towards a potential role for HCAs (Sinha *et al*, 2001, 2005c; Martinez *et al*, 2007; Ferrucci *et al*, 2009; Fu *et al*, 2011; Wang *et al*, 2011), total mutagenic activity of meat (Sinha *et al*, 2001; Fu *et al*, 2011), and B[*a*]P (Gunter *et al*, 2005; Sinha *et al*, 2005b, 2005c; Fu *et al*, 2011) in early neoplastic changes. With regard to incident adenoma, there was no association in women (RR=1.01, 95% CI=0.74–1.39) or men (RR=1.13, 95% CI=0.83–1.53) for charred meat (Tranah *et al*, 2004), suggestive positive associations with MeIQx (RR=1.28, 95% CI=0.95–1.71) and mutagenic activity (RR=1.29, 95% CI=0.97–1.72) in men (Wu *et al*, 2006), and positive associations in a mixed gender sample for PhIP and browned meat, with increased risks of 36 and 47%, respectively (Rohrmann *et al*, 2009).

As red meat was more strongly associated with rectal cancers as compared with colon cancers in a recent large prospective cohort (Cross *et al*, 2010), some of the mixed findings for colorectal adenoma may be due, in part, to the composition of cases by anatomical subsite. We observed a similar suggestive pattern of stronger associations for meat cooking-related exposures in relation to rectal adenoma. Although there is biological plausibility for variation in aetiology by anatomical site in the colorectum, not all studies have observed clear variation in meat-related risks by subsite for prevalent (Fu *et al*, 2011) or incident (Rohrmann *et al*, 2009) adenoma. As more studies evaluate meat and colorectal neoplasia, it will be important to further evaluate whether associations differ by location. Differences in risk for the various colorectal neoplasia end points could also be present if meat and potentially carcinogenic meat-related exposures are more relevant in colorectal tumour initiation or progression, or if these exposures affect risk differently in individuals in whom neoplastic changes have already occurred in the colorectum.

Thus far, haeme iron intake from meat estimated using a database of measured values has only been evaluated in two studies of colorectal adenoma, in which there were positive, but non-significant associations (risk estimates from 1.46 to 1.50) (Ferrucci *et al*, 2009; Cross *et al*, 2011), likely because of limited power, and in the NIH-AARP Diet and Health Study for colorectal cancer, in which there was a suggestive positive association with colorectal cancer (HR=1.13, 95% CI=0.99–1.29, *P*-trend=0.022) (Cross *et al*, 2010). Other studies of haeme iron and colorectal neoplasia have used rough approximations of haeme iron from meat (Lee *et al*, 2004; Chan *et al*, 2005; Larsson *et al*, 2005; Balder *et al*, 2006; Kabat *et al*, 2007), and therefore may not be comparable to results using a quantitative database. Of note, as the haeme iron database does not contain all meats, our risk estimates likely underestimate true associations in this population. As sources of non-haeme iron are generally healthy (e.g., supplements, fortified cereals, fruit juice, bread), our inverse associations for total iron and iron from supplements are not surprising. Research on dietary iron (Wurzelmann *et al*, 1996; Nelson, 2001; Kabat *et al*, 2007; Cross *et al*, 2010) or iron stores (Knekt *et al*, 1994; Herrinton *et al*, 1995; Wurzelmann *et al*, 1996; Cross *et al*, 2006) and colorectal cancer is inconclusive, and studies of prevalent or recurrent colorectal adenoma have also produced inconsistent results (Tseng *et al*, 1996, 1997, 2000; Chan *et al*, 2005; Ferrucci *et al*, 2009).

Combined nitrate and nitrite intake has been positively associated with colorectal adenoma (two-fold increased risk) (Ward *et al*, 2007), as has nitrate with colorectal cancer (16% increased risk) (Cross *et al*, 2010). Yet, another study did not observe an association with nitrite and colorectal adenoma (OR=1.05, 95% CI=0.59–1.86) (Ferrucci *et al*, 2009), and both nitrite (RR=0.74, 95% CI=0.34–1.63) and nitrate (RR=1.04, 95% CI=0.54–2.02) were not related to colorectal cancer in another (Knekt *et al*, 1999). Further support for the NOC pathway in early colorectal neoplasia comes from our suggestive findings for processed meat and haeme iron. As processed meat, much of which is red meat, contains haeme iron, nitrate, and nitrite, these compounds may act synergistically during tumourigenesis. Haeme iron has been positively associated with endogenous *N*-nitrosation (Cross *et al*, 2003), and nitrate and nitrite consumed from processed meat add to the pool of available nitrosating agents for reactions within the gastrointestinal tract. It is important to note that we did not evaluate other sources of nitrate and nitrite, and nitrate in particular is found in other foods and drinking water.

Our study had several strengths including a large number of cases and controls without distal colorectal adenoma confirmed with sigmoidoscopy screening. With detailed meat cooking and preparation information, we could evaluate multiple potentially carcinogenic meat-related components using quantitative databases. As adenoma is a largely asymptomatic condition, it should not have influenced diet in the year preceding the completion of the FFQ, thereby limiting the potential for reverse causation. Despite the relatively large sample size, our power to detect modest associations may still have been limited, especially for evaluating differences by advanced and/or multiple adenoma status and anatomical subsite. Our population was unique because we had information on colorectal cancer screening procedures for all participants; however, because those without adenoma only underwent sigmoidoscopy, we could not evaluate adenoma in the proximal colon. Although we had incident cases, as adenoma are largely asymptomatic and follow-up sigmoidoscopies occurred at defined intervals, we were not able to evaluate these relationships with time-to-event analyses. We were able to adjust for numerous potential confounders, but we did not have comprehensive information on previous history of lower gastrointestinal endoscopies. However, individuals reporting any lower gastrointestinal procedure (proctoscopy, sigmoidoscopy, barium enema, or colonoscopy) during the 3 years before study enrolment were excluded from the trial. Finally, the screening interval changed from 3 to 5 years after baseline during the course of the trial and could have impacted the type and number of adenomas detected, yet risks did not appear to vary when we stratified by screening interval.

In summary, we observed suggestive positive associations between red and processed meat, as well as grilled meat, well or very well-done meat, PhIP, haeme iron, and nitrate/nitrite and distal colorectal adenoma. Grilled meat, well or very well-done meat, PhIP, B[*a*]P, and mutagenic activity were all positively associated with rectal adenoma, highlighting potential aetiological differences in neoplasia throughout the colorectum. In contrast to the suggestive positive association we observed for haeme iron, we report inverse associations between total iron and iron from supplements and distal colorectal adenoma.

## Figures and Tables

**Table 1:** Means and proportions^a^ of baseline characteristics by red meat quartile (*N*=17 072)

	**Quartile of red meat intake**			
	**Q1**	**Q2**	**Q3**	**Q4**
**Characteristics**	***N*=4268**	***N*=4268**	***N*=4268**	***N*=4268**
Red meat (g per 1000 kcal), mean±s.d.	12.8±4.9	26.0±3.5	39.0±4.3	65.7±18.8
Age (years), mean±s.d.	62.8±5.3	62.5±5.2	62.2±5.1	61.5±4.9
Female, *n* (%)	2589 (60.7)	2190 (51.3)	1710 (40.1)	1130 (26.5)
				
*Ethnicity, n (%)*				
Non-Hispanic White	3579 (83.9)	3818 (89.5)	3877 (90.8)	3901 (91.4)
Non-Hispanic Black	178 (4.2)	120 (2.8)	102 (2.4)	100 (2.3)
Hispanic	63 (1.5)	43 (1.0)	56 (1.3)	84 (2.0)
Other	448 (10.5)	287 (6.7)	233 (5.5)	183 (4.3)
				
Education, college graduate or postgraduate, *n* (%)	1993 (46.7)	1799 (42.2)	1572 (36.8)	1333 (31.2)
Body mass index (kg m^−2^), mean±s.d.	25.7±4.3	26.7±4.4	27.6±4.5	28.5±4.7
Physical activity, 4+ hours per week, *n* (%)	1451 (34.0)	1113 (26.1)	1005 (23.6)	842 (19.7)
				
*Smoking status, n (%)*				
Never	2509 (58.8)	2439 (57.2)	2239 (52.5)	1935 (45.3)
Former	1629 (38.2)	1637 (38.4)	1789 (41.9)	1977 (46.3)
Current	130 (3.1)	192 (4.5)	240 (5.6)	356 (8.3)
				
Alcohol (g per day), mean±s.d.	9.0±25.4	10.3±22.1	11.4±22.4	11.0±19.6
Family history of colorectal cancer, *n* (%)	371 (8.7)	355 (8.3)	356 (8.3)	389 (9.1)
Non-steroidal anti-inflammatory drugs, 60 +pills per month, *n* (%)	459 (10.8)	499 (11.7)	506 (11.9)	496 (11.6)
Total energy intake (kcal per day), mean±s.d.	1934±705	2001±758	2122±796	2277±882
White meat (g per 1000 kcal), mean±s.d.	28.8±23.6	26.4±19.1	25.5±17.8	25.4±17.4
Dietary calcium (mg per 1000 kcal), mean±s.d.	530±194	488±160	458±145	413±127
Supplemental calcium (mg per day), mean±s.d.	359±414	309±395	245±351	185±304
Fibre (g per 1000 kcal), mean±s.d.	14.1±4.1	12.3±3.3	11.2±2.9	10.0±2.7

a May not sum to 100% because of missing data or rounding.

**Table 2:** Multivariate ORs and 95% CIs for meat intake (g per 1000 kcal) and incident distal colorectal adenoma

	**Any distal adenoma (*n*=1008)**		**Descending/sigmoid colon adenoma (*n*=772)**		**Rectal adenoma (*n*=263)**	
**Characteristics**	**Cases**	**OR^a^ (95% CI)**	**Cases**	**OR^a^ (95% CI)**	**Cases**	**OR^a^ (95% CI)**
*Red meat (median)*						
Q1 (13.5)	185	1.00	144	1.00	46	1.00
Q2 (26.0)	243	1.18 (0.96–1.45)	179	1.12 (0.89–1.41)	66	1.30 (0.88–1.92)
Q3 (38.7)	266	1.16 (0.94–1.44)	207	1.17 (0.92–1.48)	67	1.19 (0.79–1.79)
Q4 (60.1)	314	1.22 (0.98–1.52)	242	1.22 (0.95–1.56)	84	1.33 (0.87–2.04)
*P*-trend^b^		0.12		0.12		0.24
						
*White meat (median)*						
Q1 (9.3)	235	1.00	193	1.00	53	1.00
Q2 (17.2)	265	1.65 (0.96–1.39)	200	1.06 (0.86–1.30)	73	1.37 (0.96–1.96)
Q3 (26.8)	274	1.22 (1.02–1.48)	198	1.07 (0.87–1.32)	76	1.46 (1.01–2.09)
Q4 (45.8)	234	1.08 (0.88–1.31)	181	1.01 (0.81–1.25)	61	1.19 (0.81–1.74)
*P*-trend^b^		0.60		0.90		0.36
						
*Processed meat*^c^ *(median)*						
Q1 (1.5)	186	1.00	141	1.00	50	1.00
Q2 (4.0)	242	1.15 (0.94–1.42)	186	1.16 (0.92–1.47)	62	1.09 (0.74–1.62)
Q3 (7.6)	256	1.10 (0.89–1.36)	193	1.08 (0.84–1.38)	74	1.17 (0.78–1.74)
Q4 (15.5)	324	1.23 (0.99–1.54)	252	1.24 (0.97–1.59)	77	1.08 (0.71–1.65)
*P*-trend^b^		0.12		0.14		0.92

Abbreviations: BMI=body mass index; CI=confidence interval; NSAID=non-steroidal anti-inflammatory drug; OR=odds ratio.

a OR adjusted for age at baseline (⩽59, 60–64, 65–70, 70+ years), study centre (10 centres), gender, ethnicity (non-Hispanic White, non-Hispanic Black, Hispanic, other), education (⩽high school, some college or post-high school training, college graduate, or postgraduate), family history of colorectal cancer (yes, no, missing), BMI (<25, 25–<30, ⩾30 kg m^−2^, missing), NSAIDs use (<4, 4–29, 30–59, 60+ pills per month), physical activity (0, <1, 2, 3, 4+ hours per week), smoking status (never, former, current), alcohol intake (g per day, continuous), dietary calcium (mg per 1000 kcal, continuous), supplemental calcium (mg per day, continuous), dietary fibre (g per 1000 kcal, continuous), and total energy intake (kcal per day, continuous).

b *P*-trend calculated using the median of each quartile.

c Includes bacon, sausage, hot dogs, ham, liver, and cold cuts.

**Table 3:** Multivariate ORs and 95% CIs for meat-cooking methods (g per 1000 kcal), doneness levels (g per 1000 kcal), individual meat mutagens (ng per day), and total mutagenic activity (revertant colonies per day) in relation to incident distal colorectal adenoma

	**Any distal adenoma (*n*=1008)**		**Descending/sigmoid colon adenoma (*n*=772)**		**Rectal adenoma (*n*=263)**	
**Characteristics**	**Cases**	**OR^a^ (95% CI)**	**Cases**	**OR^a^ (95% CI)**	**Cases**	**OR^a^ (95% CI)**
*Grilled meat (median)*						
Q1 (0.0)	225	1.00	176	1.00	55	1.00
Q2 (1.5)	212	1.12 (0.91–1.38)	163	1.10 (0.88–1.40)	54	1.20 (0.80–1.79)
Q3 (6.1)	271	1.26 (1.03–1.54)	202	1.21 (0.96–1.52)	68	1.36 (0.92–2.03)
Q4 (16.4)	300	1.20 (0.97–1.49)	231	1.18 (0.93–1.51)	86	1.56 (1.04–2.36)
*P*-trend^b^		0.11		0.29		0.05
						
*Pan fried meat (median)*						
Q1 (0.3)	209	1.00	157	1.00	58	1.00
Q2 (1.3)	255	1.06 (0.87–1.29)	194	1.08 (0.86–1.34)	67	0.99 (0.68–1.43)
Q3 (3.8)	269	1.05 (0.86–1.28)	208	1.08 (0.86–1.35)	67	0.94 (0.64–1.37)
Q4 (11.7)	275	1.03 (0.83–1.28)	213	1.04 (0.81–1.34)	71	1.02 (0.68–1.54)
*P*-trend^b^		0.80		0.76		0.99
						
*Well/very well meat (median)*						
Q1 (1.0)	189	1.00	147	1.00	44	1.00
Q2 (3.0)	281	1.39 (1.14–1.69)	219	1.39 (1.12–1.74)	71	1.52 (1.03–2.24)
Q3 (6.0)	265	1.25 (1.02–1.53)	199	1.20 (0.95–1.51)	71	1.47 (0.99–2.19)
Q4 (13.6)	273	1.20 (0.95–1.49)	207	1.14 (0.89–1.46)	77	1.59 (1.05–2.43)
*P*-trend^b^		0.67		0.79		0.14
						
*DiMeIQx*						
Q1 (0.0)	233	1.00	189	1.00	54	1.00
Q2 (0.5)	244	1.02 (0.84–1.23)	188	0.96 (0.78–1.18)	59	1.07 (0.74–1.56)
Q3 (1.4)	241	0.95 (0.78–1.14)	182	0.87 (0.71–1.08)	69	1.19 (0.83–1.72)
Q4 (3.8)	288	0.99 (0.82–1.20)	213	0.89 (0.72–1.11)	79	1.20 (0.82–1.74)
*P*-trend^b^		0.85		0.34		0.38
						
*MeIQx*						
Q1 (4.8)	202	1.00	162	1.00	48	1.00
Q2 (13.1)	255	1.12 (0.92–1.36)	189	1.04 (0.83–1.29)	66	1.22 (0.83–1.79)
Q3 (26.4)	262	1.04 (0.85–1.27)	196	0.97 (0.77–1.22)	71	1.19 (0.81–1.77)
Q4 (62.5)	287	0.99 (0.80–1.23)	225	0.97 (0.76–1.24)	76	1.12 (0.74–1.72)
*P*-trend^b^		0.44		0.70		0.97
						
*PhIP*						
Q1 (10.8)	187	1.00	152	1.00	40	1.00
Q2 (36.2)	250	1.17 (0.96–1.43)	193	1.12 (0.90–1.40)	62	1.41 (0.94–2.12)
Q3 (84.3)	282	1.19 (0.97–1.46)	218	1.14 (0.91–1.42)	73	1.53 (1.01–2.30)
Q4 (234.5)	285	1.18 (0.96–1.45)	209	1.07 (0.85–1.36)	86	1.75 (1.17–2.64)
*P*-trend^b^		0.89		0.98		0.02
						
*B[a]P*						
Q1 (0.5)	225	1.00	184	1.00	50	1.00
Q2 (2.7)	253	1.01 (0.83–1.22)	193	0.93 (0.75–1.15)	64	1.15 (0.79–1.69)
Q3 (17.3)	244	1.03 (0.85–1.24)	193	0.99 (0.80–1.22)	57	1.09 (0.74–1.60)
Q4 (79.0)	284	1.06 (0.88–1.29)	202	0.92 (0.74–1.15)	90	1.53 (1.06–2.20)
*P*-trend^b^		0.46		0.60		0.02
						
*Mutagenic activity*						
Q1 (692)	200	1.00	163	1.00	42	1.00
Q2 (2146)	260	1.13 (0.93–1.37)	192	1.01 (0.81–1.26)	74	1.58 (1.07–2.34)
Q3 (4312)	249	0.97 (0.79–1.19)	195	0.92 (0.73–1.15)	58	1.13 (0.74–1.73)
Q4 (9902)	297	1.06 (0.86–1.31)	222	0.96 (0.76–1.22)	87	1.57 (1.03–2.40)
*P*-trend^b^		0.46		0.73		0.15

Abbreviations: BMI=body mass index; CI=confidence interval; DiMeIQx=2-amino-3,4,8-trimethylimidazo[4,5-*f*]quinoxaline; MeIQx=2-amino-3,8-dimethylimidazo[4,5-*f*]quinoxaline; NSAID=non-steroidal anti-inflammatory drug; OR=odds ratio; PhIP=2-amino-1-methyl-6-phenyl-imidazo[4,5-*b*]pyridine.

a OR adjusted for age at baseline (⩽59, 60–64, 65–70, 70+ years), study centre (10 centres), gender, ethnicity (non-Hispanic White, non-Hispanic Black, Hispanic, other), education (⩽high school, some college or post-high school training, college graduate, or postgraduate), family history of colorectal cancer (yes, no, missing), BMI (<25, 25–<30, ⩾30 kg m^−2^, missing), NSAIDs use (<4, 4–29, 30–59, 60+ pills per month), physical activity (0, <1, 2, 3, 4+ hours per week), smoking status (never, former, current), alcohol intake (g per day, continuous), dietary calcium (mg per 1000 kcal, continuous), supplemental calcium (mg per day, continuous), dietary fibre (g per 1000 kcal, continuous), and total energy intake (kcal per day, continuous).

b *P*-trend calculated using the median of each quartile.

**Table 4:** Multivariate ORs and 95% CIs for total iron (mg per day), dietary iron (mg per 1000 kcal), iron and haeme iron from meat (mg per 1000 kcal), iron from supplements (mg per day), and nitrate plus nitrite (mg per 1000 kcal) and incident distal colorectal adenoma

	**Any distal adenoma (*n*=1008)**		**Descending/sigmoid colon adenoma (*n*=772)**		**Rectal adenoma (*n*=263)**	
**Characteristics**	**Cases**	**OR^a^ (95% CI)**	**Cases**	**OR^a^ (95% CI)**	**Cases**	**OR^a^ (95% CI)**
*Total iron*^b^ *(median)*						
Q1 (14.0)	321	1.00	246	1.00	81	1.00
Q2 (18.9)	240	0.83 (0.68–1.00)	183	0.83 (0.67–1.02)	63	0.86 (0.60–1.23)
Q3 (30.8)	254	0.87 (0.73–1.05)	199	0.89 (0.72–1.09)	66	0.94 (0.66–1.34)
Q4 (39.0)	193	0.69 (0.56–0.86)	144	0.67 (0.52–0.86)	53	0.81 (0.54–1.23)
*P*-trend^c^		0.01		0.02		0.54
						
*Dietary iron (median)*						
Q1 (6.8)	307	1.00	236	1.00	78	1.00
Q2 (8.2)	285	1.09 (0.91–1.30)	211	1.06 (0.86–1.30)	78	1.12 (0.80–1.67)
Q3 (9.4)	211	0.85 (0.70–1.05)	171	0.92 (0.73–1.15)	50	0.76 (0.51–1.12)
Q4 (11.7)	204	0.85 (0.68–1.07)	154	0.87 (0.67–1.13)	57	0.92 (0.60–1.41)
*P*-trend^c^		0.06		0.18		0.42
						
*Iron from meat (median)*						
Q1 (0.2)	209	1.00	156	1.00	59	1.00
Q2 (0.4)	226	0.94 (0.77–1.15)	168	0.97 (0.77–1.22)	60	0.91 (0.63–1.31)
Q3 (0.7)	257	0.99 (0.81–121)	205	1.07 (0.86–1.35)	57	0.79 (0.53–1.16)
Q4 (1.0)	316	1.08 (0.88–1.33)	243	1.13 (0.89–1.43)	87	1.06 (0.72–1.56)
*P*-trend^c^		0.26		0.18		0.59
						
*Haeme iron from meat (median)*						
Q1 (0.07)	200	1.00	153	1.00	54	1.00
Q2 (0.10)	227	1.03 (0.84–1.26)	168	0.99 (0.79–1.26)	59	0.99 (0.67–1.45)
Q3 (0.20)	248	1.01 (0.82–1.24)	197	1.05 (0.83–1.32)	58	0.88 (0.59–1.30)
Q4 (0.40)	332	1.23 (0.99–1.52)	254	1.23 (0.96–1.56)	91	1.25 (0.84–1.86)
*P*-trend^b^		0.03		0.04		0.18
						
*Iron from supplements (cut points)*						
0.0	610	1.00	467	1.00	157	1.00
0.1–18.0	369	0.89 (0.77–1.03)	285	0.88 (0.75–1.04)	99	0.97 (0.73–1.28)
>18.0–72.0	29	0.65 (0.44–0.97)	20	0.58 (0.36–0.92)	7	0.65 (0.30–1.43)
*P*-trend^c^		0.02		0.02		0.46
						
*Nitrate plus nitrite (median)*						
Q1 (0.06)	186	1.00	150	1.00	44	1.00
Q2 (0.17)	227	1.09 (0.89–1.34)	165	0.98 (0.77–1.23)	64	1.31 (0.88–1.95)
Q3 (0.36)	268	1.15 (0.93–1.42)	203	1.07 (0.84–1.35)	75	1.38 (0.92–2.07)
Q4 (0.84)	327	1.22 (0.94–1.53)	254	1.16 (0.90–1.50)	80	1.27 (0.80–1.99)
*P*-trend^c^		0.14		0.15		0.72

Abbreviations: BMI=body mass index; CI=confidence interval; NSAID=non-steroidal anti-inflammatory drug; OR=odds ratio.

a OR adjusted for age at baseline (⩽59, 60–64, 65–70, 70+ years), study centre (10 centres), gender, ethnicity (non-Hispanic White, non-Hispanic Black, Hispanic, other), education (⩽high school, some college or post-high school training, college graduate, or postgraduate), family history of colorectal cancer (yes, no, missing), BMI (<25, 25–<30, ⩾30 kg m^−2^, missing), NSAIDs use (<4, 4–29, 30–59, 60+ pills per month), physical activity (0, <1, 2, 3, 4+ hours per week), smoking status (never, former, current), alcohol intake (g per day, continuous), dietary calcium (mg per 1000 kcal, continuous), supplemental calcium (mg per day, continuous), dietary fibre (g per 1000 kcal, continuous), and total energy intake (kcal per day, continuous).

b Dietary iron (residual energy adjusted) plus iron from supplements. Nutrients in this model were adjusted using residual energy adjustment.

c *P*-trend calculated using the median of each quartile or category.
